# Genetic Analysis of Patients With Common Variable Immunodeficiency Followed in the Pulmonology Department: A Report of Five Patients

**DOI:** 10.7759/cureus.107885

**Published:** 2026-04-28

**Authors:** Loubna Boumekrat, Abir Bouhamdi, Badereddine Elmakhzan, Souad Aalil, Btissame Es-Sabbahi, Lamiae Senhaji, Meryem Karhate, Mounia Serraj, Mohamed ElBiaze, Mohamed Chakib Benjelloun, Bouchra Amara

**Affiliations:** 1 Pneumology, Hassan II University Hospital, Fez, MAR; 2 Pneumology, Hassan II University Hospital, Sidi Mohamed Ben Abdellah University, Fez, MAR; 3 Medical Genetics, Faculty of Medicine and Pharmacy, Hassan II University Hospital, Fez, MAR; 4 Pulmonology and Critical Care, Hassan II University Hospital, Fez, MAR

**Keywords:** cd3g, common variable immunodeficiency (cvid), genetics, il7r, nfkb1, primary immunodeficiency

## Abstract

Common variable immunodeficiency (CVID) is a primary immunodeficiency characterized by hypogammaglobulinemia, impaired vaccine response, and increased susceptibility to infections. Advances in molecular genetics have enabled the identification of monogenic forms in an increasing proportion of patients. In this study, a targeted genetic panel based on the 2022 International Union of Immunological Societies classification was performed in five patients presenting with a phenotype compatible with CVID. A genetic abnormality was identified in two (40%) patients, including a variant in the *NFKB1* gene in one patient and variants in the *CD3G* and *IL7R* genes in another. No detectable genetic abnormalities were found in three (60%) patients. These results are consistent with the marked genetic heterogeneity of CVID and highlight the value of molecular sequencing in improving diagnostic accuracy and guiding therapeutic strategies.

## Introduction

Common variable immunodeficiency (CVID) is one of the most prevalent primary immunodeficiencies in adults [1]. It is characterized by persistent hypogammaglobulinemia, impaired vaccine responses, and increased susceptibility to bacterial infections, primarily affecting the respiratory tract. Beyond recurrent infections, a wide range of non-infectious manifestations may occur, including autoimmune, granulomatous, hepatic, and lymphoproliferative complications.

Advances in next-generation sequencing (NGS) have enabled the identification of monogenic defects in approximately 10-20% of cases [2,3]. Variants in the *NFKB1* gene are among the most frequently reported genetic causes in patients with CVID and represent the most common monogenic cause, in a context where identifiable genetic abnormalities account for approximately 10-20% of cases. [4,5]. This study aims to evaluate the contribution of a targeted genetic panel in a cohort of five patients presenting a CVID phenotype.

## Case presentation

Patient selection and clinical data

This study included five patients followed for CVID at the Department of Pulmonology, Hassan II University Hospital, Fez, Morocco. Patients were selected based on the presence of recurrent infections, particularly respiratory infections, associated with hypogammaglobulinemia. Clinical, radiological, and immunological data were retrospectively collected from medical records.

Diagnostic criteria for common variable immunodeficiency

The diagnosis of CVID was established according to the criteria of the European Society for Immunodeficiencies (ESID) and the International Consensus Document (ICON) criteria, including a significant decrease in serum IgG and IgA levels, with or without a decrease in IgM, confirmed on at least two measurements; impaired humoral response or the presence of recurrent infections; and exclusion of secondary causes of hypogammaglobulinemia, such as protein loss, immunosuppressive treatments, or hematological malignancies.

Genetic analysis

Genetic analysis was performed using NGS on the Ion Torrent platform, using a targeted panel of genes involved in primary immunodeficiencies (according to the IUIS 2022 classification). Sequencing data were analyzed using a dedicated bioinformatics pipeline including alignment to the reference genome, variant calling, and annotation. Variant interpretation was performed according to the recommendations of the American College of Medical Genetics and Genomics (ACMG). Variants were classified as pathogenic, likely pathogenic, or variants of uncertain significance (VUS). Only pathogenic and likely pathogenic variants were retained for interpretation.

Ethical considerations

This study was conducted in accordance with the principles of the Declaration of Helsinki. Given the retrospective nature of the study, patient data were anonymized. Ethical approval was obtained from the local institutional ethics committee, and informed consent was obtained from patients when required.

Case 1

A 33-year-old female patient presented with a history of recurrent respiratory infections associated with repeated ENT infections evolving over four years. The main complaint was chronic cough with purulent sputum associated with chronic dyspnea classified as stage I according to the Modified Medical Research Council (mMRC) scale (Table 1).

**Table 1:** Clinical and radiological manifestations of the patients.

Parameters	Case 1	Case 2	Case 3	Case 4	Case 5
Age at diagnosis (years)	29	26	19	29	44
Diagnostic delay (years)	4	5	14	5	6
Recurrent respiratory infections	Yes	Yes	Yes	Yes	Yes
Dyspnea (mMRC)	Stage I	Stage I	Stage III	Stage III	Stage IV
Chronic productive cough	Yes	Yes	Yes	Yes	Yes
Gastrointestinal infections	No	Yes	No	Yes	No
Recurrent ENT infections	Yes	Yes	No	Yes	Yes
Documented severe infection	No	Pyothorax	Severe pneumonia	Purulent pericarditis, pneumococcal meningoencephalitis, tubo-ovarian abscess	Tuberculosis (2023)
Autoimmune involvement	No	No	No	No	No
Hepatic involvement	No	No	No	Portal hypertension	No
Malignancy	No	No	Metastatic adenocarcinoma (death)	No	No
Chest X-ray/CT findings	Bilateral consolidation and mediastinal lymphadenopathy	Diffuse bronchiectasis	Mediastinal lymphadenopathy + chronic bilateral consolidation with traction bronchiectasis	Bilateral lower lobe consolidations	Diffuse bronchiectasis

Chest radiography revealed bilateral pneumonia. Due to persistent dyspnea, a chest CT scan was obtained, showing parenchymal consolidation of the apical segment of the left lower lobe, associated with small diffuse nodules in both lung fields and mediastinal lymphadenopathy. Pulmonary tuberculosis was initially suspected. Bronchoscopy was unremarkable, with negative direct examination and culture for acid-fast bacilli (AFB), as well as a negative GeneXpert *Mycobacterium tuberculosis* (MTB) test. Mediastinoscopy revealed a reactive lymphadenitis.

Serum protein electrophoresis showed an inflammatory pattern with increased alpha-1, alpha-2, and beta-1 globulin fractions, associated with hypogammaglobulinemia. Immunoglobulin quantification confirmed hypogammaglobulinemia (IgA = 0.25 g/L, IgG = 3.2 g/L, IgM = 0.16 g/L). Immunophenotyping showed decreased CD19+ B lymphocytes (57 cells/µL) (Table 2).

**Table 2:** Immunological profile of patients with common variable immunodeficiency.

Parameters	Case 1	Case 2	Case 3	Case 4	Case 5
IgA (g/L)	0.25	<0.01	0.25	0.05	0.25
IgG (g/L)	3.2	<0.23	3.2	1.08	3.20
IgM (g/L)	0.16	0.25	0.25	0.05	0.25
CD3+ (cells/μL)	—	1,174 (N)	1,011	—	—
CD4+ (cells/μL)	—	508 (N)	282	—	—
CD8+ (cells/μL)	—	650 (N)	706	—	—
CD19+ (cells/μL)	57	5 ↓	—	—	—
CD20+ (cells/μL)	—	7 ↓	—	—	—
CD16+, CD56+ (cells/μL)	—	99 (N)	—	—	—

No pathogenic or likely pathogenic variants were identified according to the ACMG criteria. Additionally, no VUS were identified (Table 3).

**Table 3:** Genetic findings in patients with common variable immunodeficiency.

Parameters	Case 1	Case 2	Case 3	Case 4	Case 5
Genetic findings	No pathogenic variant	*NFKB1* variant (c.533del)	No pathogenic variant	*CD3G* (c.213del) + *IL7R* (c.80del; c.82+147_82+148insC)	No pathogenic variant

The diagnosis of CVID was established according to ESID/ICON criteria, after exclusion of secondary causes of hypogammaglobulinemia. The patient was treated with almost monthly intravenous immunoglobulin infusions, with a marked reduction in infectious episodes over time.

Case 2

**Figure 1:**
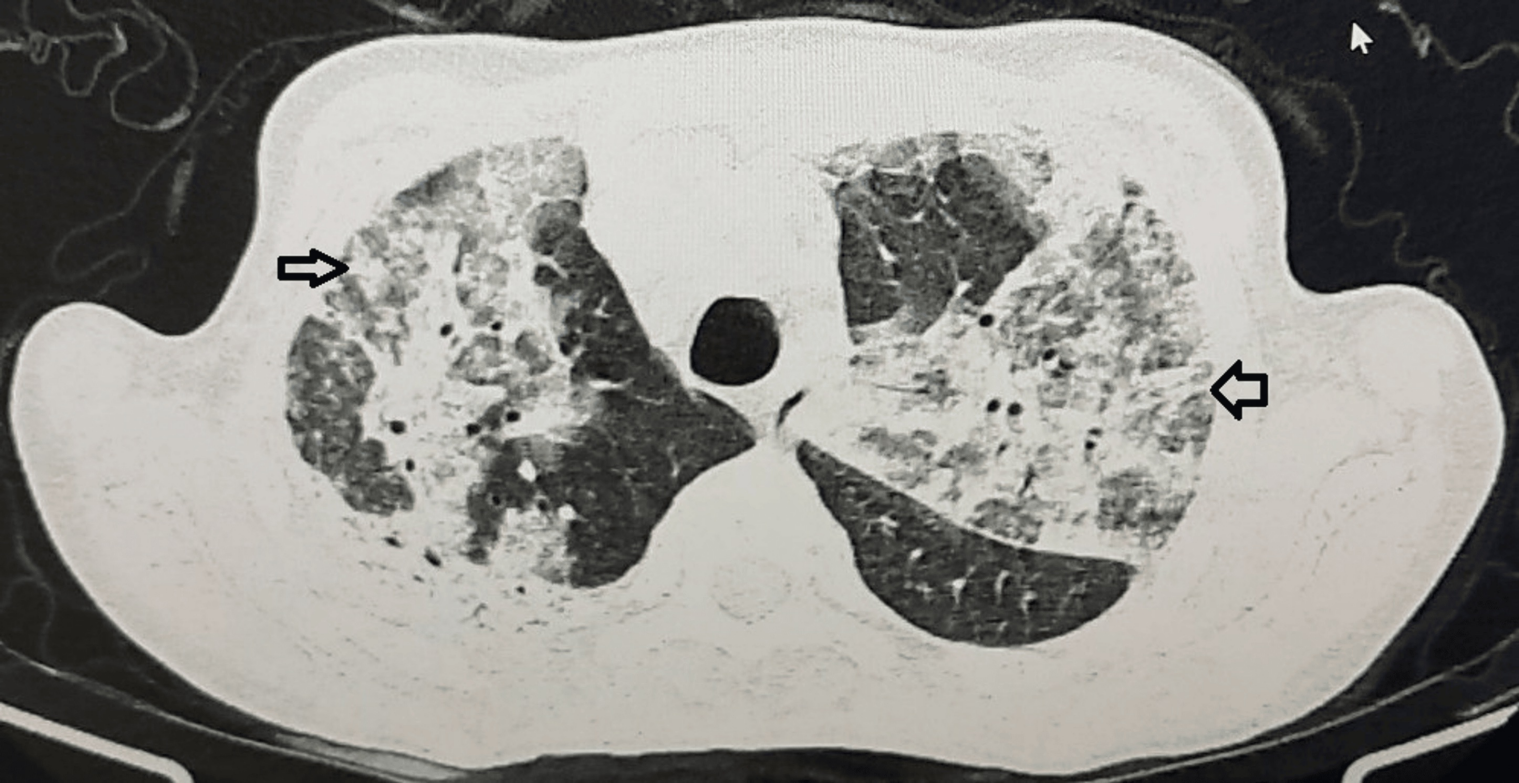
Case 2: Axial chest CT scan in the parenchymal window showing bilateral areas of consolidation associated with diffuse bronchial dilatations (bronchiectasis) in a patient with common variable immunodeficiency.

A 31-year-old female patient presented with a history of recurrent respiratory infections, chronic productive cough, repeated ENT infections, and frequent gastrointestinal infections (>4 episodes/year) (Table 1). The patient also had a history of pyothorax requiring chest drainage and antibiotic therapy, with a favorable outcome. A few days later, she presented with acute worsening of dyspnea and productive cough. Chest CT scan revealed bilateral pneumonia associated with diffuse bronchiectasis (Figure 1).

Immunological workup showed severe hypogammaglobulinemia (IgA < 0.01 g/L, IgG < 0.23 g/L, IgM = 0.25 g/L). Immunophenotyping demonstrated marked B-cell lymphopenia (CD19+ = 5 cells/µL, CD20+ = 7 cells/µL) (Table 2). Genetic testing identified a variant in the *NFKB1* gene (NM_003998.4:c.533del) (Table 3).

The diagnosis of CVID was established according to ESID/ICON criteria, after exclusion of secondary causes of hypogammaglobulinemia. The patient was started on monthly intravenous immunoglobulin infusions combined with respiratory physiotherapy, with a significant decrease in infectious episodes.

Case 3

A 43-year-old male patient presented with a history of pleuropericarditis of unknown etiology and severe acute pneumonia due to multidrug-resistant *Staphylococcus aureus* (treated). Pulmonary tuberculosis was suspected based on a constellation of clinical and radiological findings, leading to the initiation of empirical treatment before microbiological confirmation. However, subsequent investigations (AFB smear and MTB polymerase chain reaction) remained negative, rendering this diagnosis unconfirmed (Table 1).

**Figure 2:**
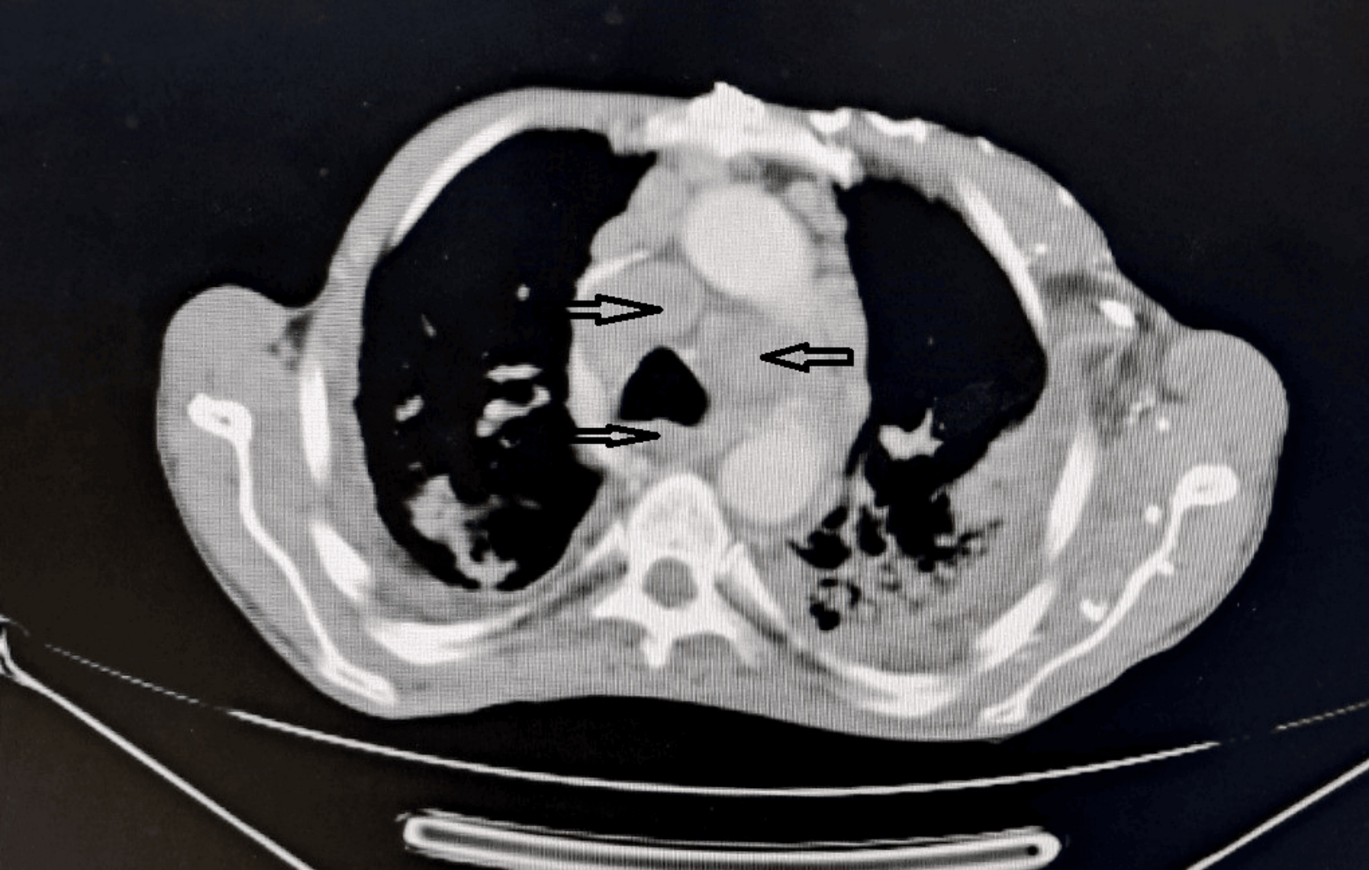
Case 3: Chest CT in axial section, mediastinal window, showing mediastinal lymphadenopathy in a patient who developed pancreatic adenocarcinoma.

**Figure 3:**
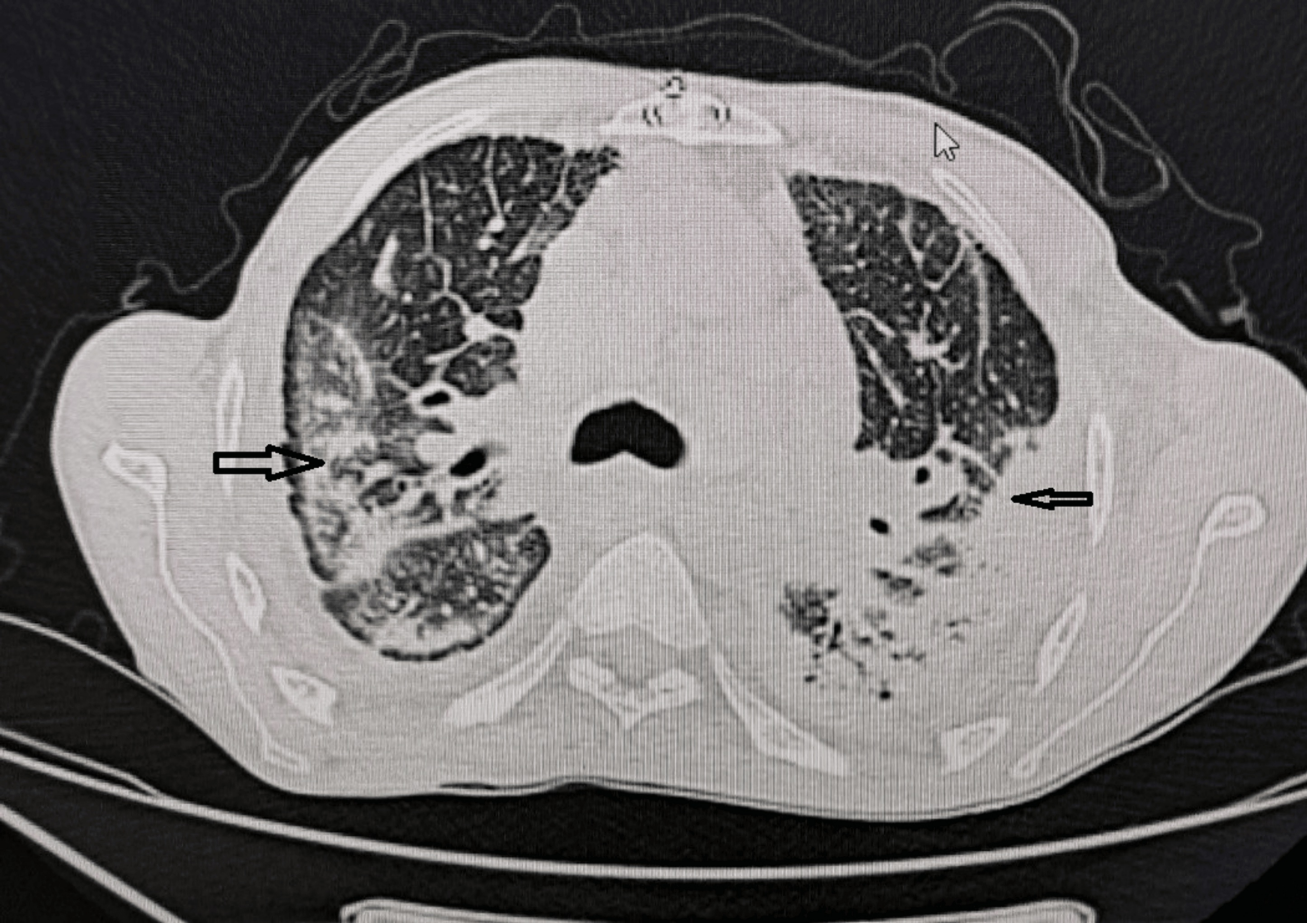
Case 3: Chest CT in axial section, parenchymal window, showing extensive pneumonic consolidations in a patient with common variable immunodeficiency, associated with bilateral bronchiectasis.

The patient presented with a chronic productive cough associated with stage III dyspnea according to the mMRC scale (Table 1), in the context of weight loss and asthenia. Clinical examination revealed exertional desaturation and cervical lymphadenopathy. A chest CT scan showed bilateral consolidations, traction bronchiectasis, and mediastinal lymphadenopathy (Figures 2, 3).

Bronchoscopy was normal, with negative AFB testing and negative GeneXpert MTB. Immunological evaluation showed hypogammaglobulinemia (IgA = 0.25 g/L, IgG = 3.2 g/L, IgM = 0.25 g/L). Immunophenotyping revealed CD3+ = 1011 cells/µL, CD4+ = 282 cells/µL (decreased), and CD8+ = 706 cells/µL (Table 2).

Cervical lymph node biopsy revealed adenocarcinoma of probable biliodigestive origin (the patient died before further gastrointestinal evaluation). The patient received urgent intravenous immunoglobulin therapy; however, the outcome was rapidly fatal due to severe clinical deterioration despite palliative oncologic care. No genetic abnormality was identified (Table 3).

Case 4

**Figure 4:**
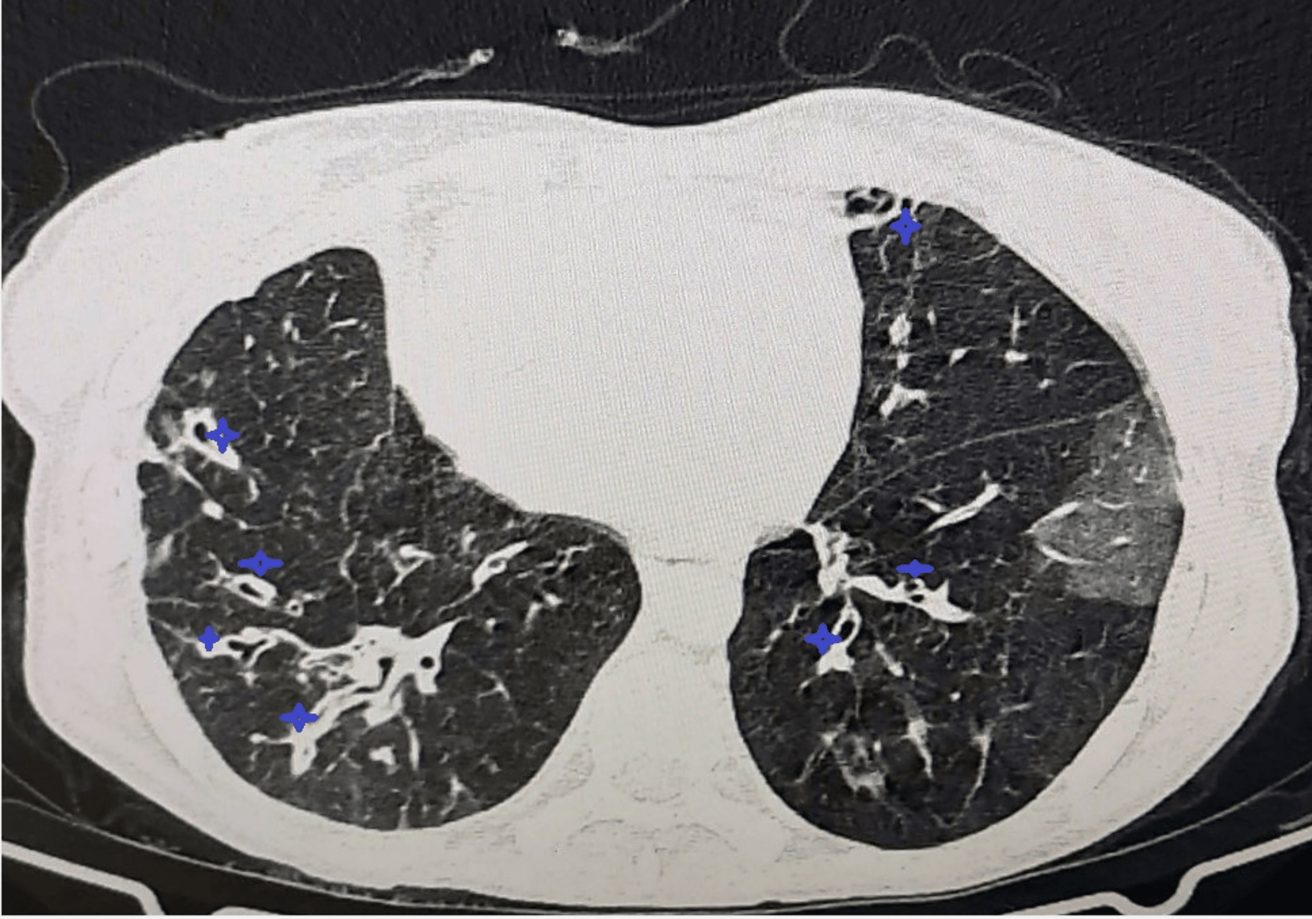
Case 4: Chest CT scan, axial section in parenchymal window, showing extensive pneumonic consolidations in a patient with common variable immunodeficiency, associated with bilateral bronchiectasis.

A 34-year-old female patient presented with a history of recurrent respiratory and ENT infections, associated with episodes of diarrhea, pneumococcal meningoencephalitis, recurrent otitis and sinusitis, portal hypertension, and a tubo-ovarian abscess complicated by peritoneal irritation (Table 1). The main reason for admission was acute respiratory failure. A chest CT scan showed bilateral infected bronchiectasis (Figure 4).

Immunological evaluation revealed hypogammaglobulinemia (IgA = 0.05 g/L, IgG = 1.08 g/L, IgM = 0.05 g/L), confirming the diagnosis of CVID (Table 2). Genetic analysis identified variants in the *CD3G* gene (NM_000073.3:c.213del) and *IL7R* gene (NM_002185.5:c.80del; c.82+147_82+148insC) (Table 3).

The patient was treated with almost monthly intravenous immunoglobulin infusions (depending on drug availability), combined with respiratory physiotherapy, leading to a reduction in infectious episodes.

Case 5

A 50-year-old female patient had a history of recurrent pneumonia and repeated ENT infections since the age of 35. She presented with acute dyspnea associated with a purulent productive cough. The workup concluded to severe acute pneumonia complicated by acute respiratory failure secondary to superinfection of diffuse bronchiectasis (Table 1). Emergency management included non-invasive ventilation, respiratory physiotherapy, and antibiotic therapy.

CVID was initially suspected and subsequently confirmed according to the ESID criteria, based on significant hypogammaglobulinemia (IgA = 0.25 g/L, IgG = 3.2 g/L, IgM = 0.45 g/L) associated with recurrent infections (Table 2), after rigorous exclusion of secondary causes, including protein loss, immunosuppressive treatments, and hematologic disorders.

The patient was started on almost monthly intravenous immunoglobulin infusions, combined with respiratory physiotherapy and home oxygen therapy. A reduction in infectious episodes was observed. No genetic abnormality was identified (Table 3).

## Discussion

CVID is one of the most frequent primary immunodeficiencies in adults, characterized by impaired humoral immunity combining hypogammaglobulinemia and increased susceptibility to infections, particularly respiratory infections [1]. Its diagnosis is based on international criteria (ICON/ESID), including a significant decrease in IgG and IgA, with or without reduced IgM, confirmed on at least two measurements, as well as the exclusion of secondary causes of hypogammaglobulinemia [2,3].

In our series, a female predominance was noted (four women and one man, Case 3), consistent with some literature data [1,2]. The mean age at diagnosis was 30 years, in agreement with data placing diagnosis between the second and fourth decades of life [3]. Clinically, recurrent respiratory infections were present in all patients, combining chronic productive cough and repeated episodes of pneumonia. This presentation corresponds to the most frequent CVID phenotype [3]. The prolonged course of these infections led to the development of diffuse bronchiectasis in three patients (Cases 2, 3, and 4), reflecting the progressive and destructive nature of the disease in the absence of early management [3].

ENT manifestations were observed in four patients (Cases 1, 2, 4, and 5), dominated by recurrent sinusitis and upper respiratory tract infections. Digestive involvement was found in two patients (Cases 2 and 4), in the form of chronic gastrointestinal symptoms, consistent with digestive manifestations described in CVID [3].

A history of severe infections was reported in four patients: one pyothorax (Case 2), one severe pneumonia (Case 3), and multiple severe infections in Case 4 (pneumococcal meningitis, purulent peritonitis, and tubo-ovarian abscess). These findings illustrate the increased susceptibility to invasive infections in CVID [3].

Case 3 also illustrated the occurrence of a probable biliodigestive adenocarcinoma during CVID, a known complication related to defective immune surveillance. Although lymphomas are the most frequent, an increased incidence of solid tumors, particularly digestive cancers, has been described in the literature [1,3]. This observation, derived from a single case (Case 3), suggests a possible association and highlights the importance of clinical vigilance regarding neoplasms in these patients, requiring confirmation in larger-scale studies.

The diagnostic delay ranged from four years (Case 1) to 14 years (Case 3). The case with the longest delay (Case 3) was also associated with more severe pulmonary involvement, suggesting the impact of delayed diagnosis on prognosis, particularly respiratory outcomes [1,2].

Differential diagnosis was systematically considered and ruled out in all patients, particularly secondary causes of hypogammaglobulinemia, including lymphoid malignancies, protein loss, and iatrogenic causes, in accordance with international recommendations [2,3].

The diagnosis of CVID was confirmed in all patients by immunological workup, showing hypogammaglobulinemia with decreased IgG and IgA in all five cases. A decrease in IgM was observed in two patients (Cases 1 and 4). These results are consistent with international diagnostic criteria for CVID [1]. A decrease in B lymphocytes was found in Cases 1 and 2, while a decrease in CD4+ lymphocytes was observed in Case 3.

Genetic results and therapeutic implications

Genetic analysis using a panel dedicated to CVID (Table 4) identified molecular abnormalities in two patients (2/5 cases).

**Table 4:** Classification of studied genes according to 2022 International Union of Immunological Societies classification.

IUIS 2022 category	Gene	Main function/Pathophysiological role
Combined immunodeficiencies (CIDs)	ADA	Purine metabolism; lymphotoxicity affecting T, B, and NK cells
	IL2RG	Common γ chain; essential for T and NK cell development
	JAK3	Cytokine signaling; lymphopoiesis
	RAG1	V(D)J recombination; T and B cell receptor diversity
	RAG2	Works with RAG1 in antigen receptor maturation
	DCLRE1C (Artemis)	DNA repair; radiosensitive CID
	CD3D	Component of the TCR-CD3 complex; T cell activation
	CD3E	TCR signaling; T cell maturation
	CD3G	TCR expression and signaling
	PNP	Purine metabolism; selective T cell toxicity
Combined immunodeficiencies with syndromic features (CID-SF)	ATP6AP1	Organelle acidification; immune and hepatic involvement
	BTK	B cell maturation; X-linked agammaglobulinemia
	CD40	B cell activation; class-switch recombination
	CD40LG	T-B interaction; hyper-IgM syndrome
	DOCK8	Antiviral immunity; severe cutaneous infections
	GATA2	Hematopoiesis; innate immunity
	LCK	TCR signaling
	MALT1	NF-κB activation
	MAP3K14 (NIK)	Non-canonical NF-κB pathway regulator
	MSN	Cytoskeleton; lymphocyte migration
	NFKB1	Transcription factor; frequently involved in CVID
	NFKB2	B-cell maturation
	PIK3CD	PI3K signaling; lymphocyte activation
	PIK3R1	PI3K regulatory subunit
	PTPRC (CD45)	Major regulator of lymphocyte signaling
Predominantly antibody deficiencies (PADs)	AICDA	Class-switch recombination
	CD19	B-cell activation co-receptor
	ICOS	T-B cell cooperation
	IGHM	Primary humoral immunity
	IGLL1	Pre-BCR component; early B cell development
	UNG	Immunoglobulin diversification
Diseases of immune dysregulation (ID)	CTLA4	Major negative regulator of lymphocyte activation
	FERMT3	Leukocyte adhesion
	CORO1A	Cytoskeleton regulation; lymphocyte survival
	RFXANK	MHC class II expression
	TRAC	T cell receptor component
Congenital defects of phagocytes (PHGs)	FERMT3	Leukocyte adhesion deficiency
	CORO1A	Immune cell migration and function
Autoinflammatory diseases (AIDs)	MVK	Cholesterol metabolism; autoinflammatory syndromes
Complement deficiencies (CDs)	C1QB	Classical complement pathway activation
	C5	Membrane attack complex formation
	C6	MAC assembly
	C9	Terminal component of MAC

A mutation in the *NFKB1* gene was identified in Case 2 (Table 3), consistent with literature data describing this gene as one of the most frequent monogenic causes of CVID, involved in the regulation of the NF-κB pathway and B-cell survival [4,5].

In Case 4, variants in the *CD3G* (c.213del) and *IL7R* (c.80del; c.82+147_82+148insC) genes were identified, suggesting a combined immunodeficiency phenotype involving both B- and T-cell compartments; however, this does not fulfill the formal diagnostic criteria for combined immunodeficiency [6].

The identification of these abnormalities confirms the genetic heterogeneity of CVID and allows better pathophysiological characterization of the disease [6,7]. Molecular characterization may also guide targeted therapeutic approaches.

As general considerations, certain monogenic defects, particularly *CTLA4* and *LRBA* deficiencies, may benefit from targeted therapies such as CTLA4 fusion proteins (abatacept, belatacept). Similarly, activating mutations in *PIK3CD* may be amenable to inhibitors of the PI3K/AKT/mTOR pathway, such as sirolimus or selective PI3Kδ inhibitors. However, these abnormalities were not identified in the cases studied [6,8].

Treatment was based on lifelong immunoglobulin replacement therapy, administered intravenously or subcutaneously depending on availability. This treatment is the cornerstone of CVID management, with a need for individualized dose adjustment [1-3,6]. The therapeutic protocol included regular monthly infusions or subcutaneous injections aimed at maintaining protective IgG levels and reducing infectious complications. Respiratory physiotherapy also represents an essential component of adjunctive treatment in patients with bronchiectasis. It helps improve bronchial secretion drainage, reduce congestion, and preserve long-term respiratory function.

Thus, in our series, a trend toward more severe pulmonary involvement, particularly a higher frequency of bronchiectasis, was observed in patients with identified genetic abnormalities. However, given the small sample size (n = 2), this observation should be interpreted with caution and considered hypothesis-generating. It, nevertheless, suggests that a subgroup of patients may exhibit a more severe respiratory phenotype, warranting confirmation in larger-scale studies.

This study remains limited by a small sample size, the absence of systematic functional and familial analyses, and the use of a targeted genetic panel rather than whole-exome sequencing.

## Conclusions

This study highlights the extreme genetic heterogeneity of CVID. The identification of monogenic abnormalities in some patients highlights the value of molecular investigation, particularly in severe or atypical presentations. Our results suggest that CVID represents a heterogeneous spectrum, encompassing both genetically determined inborn errors of immunity as well as acquired and/or idiopathic forms, rather than a single entity. Overlap between humoral and combined immunodeficiencies is common, and genetic approaches should be broad and mechanistic. Multicenter studies, including whole-exome or whole-genome sequencing combined with functional analyses, will be necessary to better understand the mechanisms underlying undetectable forms and refine precision medicine approaches in CVID.
